# Dual Engines of Adsorption and Biodegradation: Ammonium Nitrogen Removal and Mechanism Analysis by EM-Modified Corn Straw Biochar in Aqueous Solution

**DOI:** 10.3390/microorganisms14091976

**Published:** 2026-09-07

**Authors:** Penghui Wu, Zijie Sang, Ge Zhang

**Affiliations:** School of Hydraulic and Electric Power, Heilongjiang University, No. 74 Xuefu Road, Nangang District, Harbin 150080, China

**Keywords:** effective microorganisms (EM), corn straw biochar, ammonium nitrogen adsorption, agricultural water quality, sustainable waste management, biochar-microbe composite

## Abstract

Agricultural ammonium pollution from farmland drainage and low-value crop straw utilization are two critical rural environmental problems that cannot be solved by single remediation approaches. Herein, a novel composite was prepared by immobilizing effective microorganisms (EM) on corn straw biochar to construct a synergistic adsorption–biodegradation system, and its nitrogen removal mechanism was systematically investigated at structural and molecular levels. Metagenomic analysis detected a complete set of heterotrophic nitrification–aerobic denitrification (HN-AD) functional genes (amoA, hao, napA, nirK, norB, nosZ) in the isolated strain *Bacillus thuringiensis* A1, revealing the genetic potential of this strain for ammonium biodegradation. EM modification optimized biochar pore structure and increased the equilibrium adsorption capacity to 1.215 mg/g, which was 66.4% higher than that of pristine biochar (0.73 mg/g). Sterilization control tests indicated that physicochemical adsorption occupied the dominant position in ammonium removal, while microbial biodegradation acted as an auxiliary removal pathway. Importantly, the synergistic relationship between the two pathways should be interpreted cautiously, since autoclaving may subtly alter biochar physicochemical properties, and direct paired characterization of viable composites before and after sterilization is technically unavailable. Kinetic and thermodynamic results further validated the improved adsorption performance after modification. Overall, EM immobilization promoted ammonium adsorption via pore optimization, while pore-confined microbes achieved sustainable HN-AD biotransformation, jointly realizing synergistic nitrogen removal. This study provides a mechanistic reference for the optimized design and application of biochar–microbe composites in agricultural nitrogen pollution control.

## 1. Introduction

In Chinese agricultural production, excessive use of nitrogen fertilizers has caused severe non-point source pollution. Ammonium nitrogen, a major nitrogen loss form from agricultural systems, is widely present in farmland drainage and livestock wastewater, threatening aquatic ecosystems and human health [1]. China generates approximately 870 million tons of straw-type agricultural waste each year. Thus, achieving a green “treating waste with waste” strategy—using crop residues to mitigate nitrogen losses from farmland—has become an important research goal for sustainable agriculture.

Conventional techniques for aqueous ammonium nitrogen removal mainly include physical [2], chemical [3], and biological methods [4], whereas these approaches generally suffer from high energy consumption, poor environmental adaptability, and secondary pollution risks. As a multifunctional environmental material with soil improvement, nutrient retention, and water purification capacities, biochar has attracted extensive attention in the field of agricultural pollution remediation. At present, numerous large-scale prevention and control technologies have been developed for agricultural non-point source pollution, and the resource utilization of farmland drainage in the Sanjiang Plain serves as a typical practical case [5]. Previous studies have verified that the adsorption of ammonium nitrogen onto corn straw biochar conforms to the pseudo-second-order kinetic model, and the adsorption process is predominantly governed by monolayer chemisorption [6]. Kohira et al. reported that biochar pyrolyzed at 400 °C exhibits the optimal ammonium nitrogen adsorption performance under alkaline conditions [7]. In addition, chemical modification approaches including potassium permanganate [8], hydrogen peroxide [9], and acid–base treatment can effectively improve the adsorption capacity of biochar [10]. Apart from its excellent adsorption performance, field application of biochar can ameliorate soil physicochemical properties, improve nutrient use efficiency, and reduce nitrogen leaching loss. A research team from the University of Western Australia modified eucalyptus biochar with nitric acid and sodium carbonate, increasing the cation exchange capacity of biochar by 15 times and doubling its ammonium nitrogen adsorption capacity [11]. The saturated spent biochar can be further recycled and reused as a slow-release fertilizer. Gezahegn et al. found that the application of water hyacinth biochar at a rate of 20 t/hm increased soil ammonium content by approximately 60% and improved maize biomass by 47.7–78.4%. These findings fully demonstrate the promising application potential of biochar in improving farmland soil fertility and crop productivity [12].

Integrating functional microorganisms with biochar is a key strategy for improving ammonium removal efficiency [13], especially under low C/N ratios and low-temperature conditions, where conventional biological methods struggle to perform. Miao et al. immobilized cold-tolerant bacteria on biochar, achieving 96.56% ammonium removal, 6.4 times that of free bacteria [14]. Tang et al. [15] applied biochar-immobilized EM bacteria to eutrophic lake water-sediment systems, achieving simultaneous removal of nitrogen, phosphorus, and heavy metals. Zhang et al. [16] developed a Fe/Mn-modified biochar-Pseudomonas SK-4 system, attaining 99.6% ammonium nitrogen and 99.43% copper removal in real wastewater. Bi et al. [17] added iron ore and biochar to low C/N ratio agricultural runoff, significantly enriching ammonia-oxidizing, heterotrophic denitrifying, and iron-ammonia-oxidizing bacteria. In summary, biochar-immobilized microbial technology exhibits promising application prospects in reducing nitrogen loss from agricultural drainage and improving nitrogen use efficiency in crop production systems.

Effective microorganisms (EM) show great promise for decentralized pollution control because of their high diversity and environmental adaptability [18]. El-Nakieb et al. reported >96% ammonium removal from textile wastewater [19]. Chen et al. isolated strain EM-H8 from a biogas digester, achieving an ammonium removal rate of 5.94 mg/L/h and converting 77.88% of initial nitrogen to N_2_ [20]. Ding et al. isolated *Bacillus thuringiensis* EM-A1, which removed 97.13% of 1000 mg/L ammonium nitrogen with only 0.91 mg/L nitrite accumulation [21]. Wang et al. confirmed that this strain carries key genes (*hao*, *norB*) [22]. Abdel-Rahim et al. demonstrated EM bacteria’s potential in high-salinity aquaculture water by improving groundwater quality and reducing heavy metal accumulation [23]. The demonstrated robustness and genetic versatility of EM strains make them particularly attractive for field-scale applications in agricultural drainage treatment and soil nitrogen management.

Numerous studies have investigated the ammonium nitrogen removal performance of single biochar or effective microorganisms (EM), and a limited number of studies have explored their combined application in water treatment. However, the application of biochar–microbe composites for intercepting nitrogen loss in agricultural systems remains insufficiently explored. Two key scientific issues still require in-depth investigation under agricultural application scenarios.

First, the respective contributions of adsorption and biodegradation to ammonium nitrogen removal by biochar–microbe composites have not been systematically quantified under field-relevant conditions of agricultural drainage and soil environments. Although the synergistic adsorption–biodegradation effect has been widely recognized, systematic control experiments that accurately distinguish these two removal pathways are still lacking. Existing relevant studies usually compare treatment performance before and after sterilization, but most of them suffer from inadequate experimental design and insufficient statistical verification. It remains unclear whether biochar modification primarily enhances adsorption capacity or stimulates microbial metabolic activity, which is essential for guiding the optimized design of biochar-based strategies for agricultural nitrogen management.

Second, the internal relationship between the pore structure evolution of modified biochar and the molecular mechanisms of microbial nitrogen removal is rarely reported in agricultural research. Current studies exhibit obvious disciplinary segmentation: material-oriented research mainly focuses on pore structure characterization, while microbiological studies concentrate on strain screening and functional gene identification. Integrated analyses combining structural optimization and microbial functional regulation are still scarce. Nevertheless, the core synergistic mechanism follows a clear logical framework: optimized pore structures not only improve the adsorption capacity of biochar directly but also enhance mass transfer efficiency, thereby constructing favorable microhabitats for immobilized microorganisms and improving substrate availability. Meanwhile, continuous microbial biodegradation provides an independent nitrogen removal pathway complementary to physical and chemical adsorption. The coexistence and mutual promotion of the two pathways constitute the essential synergistic advantage, which is particularly valuable for long-term nitrogen retention in agricultural drainage and sustained slow-release fertilization in farmland soil. To date, systematic empirical evidence that fully validates the causal chain of “pore structure optimization → improved mass transfer → enhanced microbial metabolism → promoted nitrogen removal efficiency” under agricultural conditions is still absent.

This study focuses on fundamental mechanistic exploration rather than practical engineering verification. To eliminate the interference of complex substrates in actual water environments on the synergistic adsorption–biodegradation mechanism and thereby accurately reveal the intrinsic structure–performance relationship of materials and the molecular basis of microbial nitrogen removal, all batch adsorption experiments were conducted in standard solutions prepared with deionized water. Within this research framework, this study aims to accomplish the following: (i) fabricate EM-modified corn straw biochar and systematically characterize its structural evolution and ammonium adsorption behaviors; (ii) elucidate the molecular basis of the nitrogen removal capacity of EM strains via metagenomic sequencing; and (iii) quantitatively distinguish the respective contributions of physicochemical adsorption and microbial biodegradation through sterilization control experiments. The clarified synergistic mechanism provides a theoretical foundation for subsequent applied research targeting real agricultural wastewater. Nevertheless, the adsorption capacity and biodegradation contribution reported in this work are obtained from simplified aqueous substrates, and their practical remediation performance in authentic farmland drainage and livestock wastewater requires further field validation.

## 2. Materials and Methods

### 2.1. Raw Materials and Reagents

Corn straw used in this experiment was collected from farmlands in Heilongjiang Province, China. Commercial EM stock solution, brown sugar, ammonium chloride, and all inorganic salt reagents were of analytical grade and purchased from Sinopharm Chemical Reagent Co., Ltd. Shanghai, China. Deionized water was adopted as the aqueous medium throughout all batch experiments.

### 2.2. Preparation of Pristine Biochar and EM Modification

**Figure 1:**
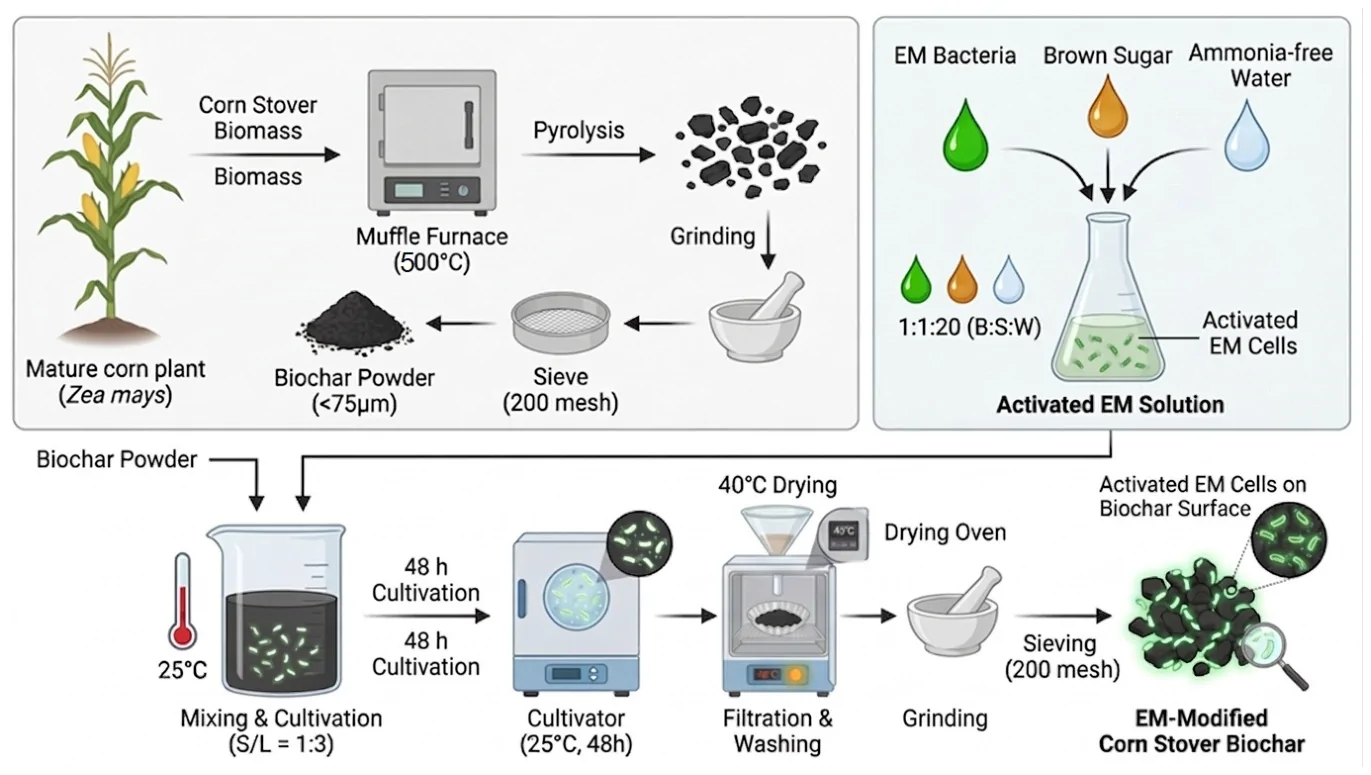
Schematic diagram of the preparation of EM-modified corn stover biochar.

Corn straw was air-dried and crushed, followed by sieving through a 100-mesh standard sieve. The sieved biomass was pyrolyzed in a muffle furnace at 500 °C under oxygen-limited conditions for 2–3 h. The pyrolytic products were repeatedly rinsed with deionized water until the filtrate reached neutral pH, then oven-dried and ground to pass through a 200-mesh sieve to obtain pristine corn straw biochar [24]. Commercial EM stock solution, brown sugar, and ammonia-free water were mixed at a volume ratio of 1:1:20 under dark conditions with stirring for microbial activation. The activated EM suspension was blended with pristine biochar at a solid–liquid ratio of 1:3 and incubated statically at 25 °C in the dark for 48 h. After incubation, the composite was thoroughly washed with deionized water to remove unimmobilized free bacteria on the surface and then dried in a forced-air oven at 40 °C for 24 h to yield EM-modified biochar [25]. The complete preparation procedure is illustrated in Figure 1.

In this study, mature modification parameters widely recognized in relevant literature were adopted. Given that the core objective of this work is to elucidate the coupled adsorption–biodegradation mechanism rather than to screen the optimal preparation process, gradient experiments were not conducted for cultivation time, EM dosage, and solid–liquid ratio. The selected parameters (solid–liquid ratio 1:3, cultivation for 48 h, drying at 40 °C) are representative and enable stable and reproducible preparation of composites for mechanistic investigation. In future research focusing on process scale-up and field application, systematic optimization of the above preparation variables will be carried out.

### 2.3. Degradation Characteristics and Genomic Analysis of EM Bacteria

Strain A1 was isolated from commercial EM microbial inoculant by serial dilution and plate-screening and selected as a representative isolate due to its excellent ammonium nitrogen removal performance. Although the commercial EM consortium comprises diverse microbial species, genomic analysis was performed on strain A1 to unravel the genetic basis for the heterotrophic nitrification-aerobic denitrification (HN-AD) metabolic potential within the EM-derived microbial community.

Degradation experiments were carried out in mineral salt medium (MSM). The MSM contained 3.8 g/L Na_2_HPO_4_, 1.0 g/L KH_2_PO_4_, 3.0 g/L KCl, 0.2 g/L MgSO_4_·7H_2_O, and 1.0 g/L NaNO_3_. The trace element solution consisted of 0.05 g/L FeSO_4_·7H_2_O, 0.01 g/L MnSO_4_·H_2_O, 0.01 g/L ZnSO_4_·7H_2_O, 0.001 g/L CuSO_4_·5H_2_O, and 0.001 g/L CoCl_2_·6H_2_O. Sodium citrate was applied as the carbon source with a fixed molar C/N ratio of 8:1, and NH_4_Cl was used as the sole ammonium nitrogen source. The initial pH was adjusted to 7.0 ± 0.1 using 1.0 M HCl or NaOH, and all media were autoclaved at 121 °C for 20 min before use.

Batch experiments were performed in 250 mL Erlenmeyer flasks containing 100 mL MSM, 1 mL bacterial suspension (inoculated from an overnight culture with the initial OD_600_ adjusted to 0.10 ± 0.02), 2 mL NH_4_Cl solution at different concentrations, and 1 mL trace-element solution. The effects of temperature (15–35 °C), initial pH (4–10), and initial ammonium concentration (100–1000 mg/L) on the degradation performance of strain A1 were investigated under continuous shaking at 150 rpm for 48 h. Samples were collected at intervals of 0, 6, 12, 18, 24, 36, and 48 h. After centrifugation at 8000 rpm for 10 min and filtration through a 0.45 μm membrane, the residual ammonium nitrogen concentration and OD_600_ were measured to evaluate nitrogen-removal efficiency and bacterial growth. Ammonium nitrogen was quantified by the Nessler’s reagent colorimetric method at 420 nm, which specifically detects NH_4_^+^-N. Accordingly, the term “ammonium removal” reported in this study refers to the decrease in aqueous NH_4_^+^-N concentration, rather than the complete mineralization of total nitrogen.

Metagenomic sequencing of strain A1 was conducted on the DNBSEQ-T7 platform. After quality control and assembly, non-redundant genes were annotated using NR, KEGG, COG, and CARD databases. Genes involved in ammonium metabolic pathways were further screened and analyzed.

### 2.4. Evaluation of Biochar Adsorption Performance

To evaluate the adsorption performance and long-term stability of the modified biochar in decentralized water purification facilities, adsorption kinetics, isothermal adsorption, and thermodynamic experiments were conducted. All adsorption batch tests were carried out using ammonium standard solutions prepared with deionized water only. Typical coexisting substances in actual agricultural wastewater, such as Ca^2+^, Mg^2+^, and humic acid were not incorporated into the experimental system. This simplified matrix was deliberately chosen to isolate the intrinsic adsorption–biodegradation mechanism from confounding environmental factors. Moreover, dynamic column tests and multiple adsorption–desorption cycle experiments were not arranged. The lack of data supporting practical engineering applications constitutes an inherent limitation of the present study.

#### 2.4.1. Adsorption Kinetics Experiment

Raw and modified biochar (1.0 g each) were accurately weighed, 50 mL of ammonium nitrogen solution with a concentration of 50 mg/L were added, and the mixture was shaken at 25 °C and 200 rpm. Samples were collected at different time points (10–180 min) to determine the remaining ammonium nitrogen concentration and calculate the adsorption capacity [26]. The data were fitted using pseudo-first-order kinetics, pseudo-second-order kinetics, and the intraparticle diffusion model to analyze the adsorption mechanism [27]. Among them, *q_t_* and *q_e_* are the adsorption amounts at time *t* and at equilibrium, respectively (mg/g); *K*_1_, *K*_2_, and *K_i_* are the rate constants corresponding to the respective models; and *C* is a constant related to the thickness of the boundary layer.

Quasi-first-order kinetic model:

(1) $$q_{t}=q_{e}\left(1-e^{-k_{1}t}\right)$$

Quasi-second-order kinetic model:

(2) $$q_{t}=\left(k_{2}q_{e}^{2}t\right)/\left(1+k_{2}q_{e}t\right)$$

Intragranular diffusion model:

(3) $$q_{t}=K_{i}t^{0.5}+C$$

#### 2.4.2. Isothermal Adsorption Experiment

The original and modified biochar samples (1.0 g each) were weighed, 50 mL of ammonium nitrogen solutions with concentrations of 10, 20, 50, 100, and 200 mg/L were added, and the mixture was shaken at 25 °C and 200 rpm until adsorption equilibrium was reached. The equilibrium concentration was determined, and the equilibrium adsorption capacity, *q_e_*, was calculated. The data were fitted separately using the Langmuir (Equation (4)) and Freundlich (Equation (5)) models to investigate the adsorption characteristics [28]. In the Langmuir model, qm represents the theoretical maximum adsorption capacity (mg/g), and *K_L_* is the affinity constant (L/mg). In the Freundlich model, *K_F_* is the adsorption capacity constant, and 1/n represents the adsorption intensity of the adsorbent.

Langmuir Equation:

(4) $$q_{e}\equiv \left(q_{max}K_{L}C_{e}\right)/\left(1+K_{L}C_{e}\right)$$

Freundlich Equation:

(5) $$q_{e}=K_{F}c_{e}^{1/n}$$

#### 2.4.3. Adsorption Thermodynamics Experiment

The original and modified biochar samples (1.0 g each) were weighed separately, and 50 mL of ammonium nitrogen solution with a concentration of 50 mg/L were added [26]. The samples were shaken at 200 rpm until equilibrium at 15, 25, and 35 °C (288.15, 298.15, and 308.15 K). The equilibrium concentration and adsorption amount were then determined. The Gibbs free energy change (*ΔG*), enthalpy change (*ΔH*), and entropy change (*ΔS*) were calculated using thermodynamic formulas by examining the adsorption amounts of ammonium nitrogen by the two types of biochar at different temperatures, revealing the patterns of the adsorption process and introducing the thermodynamic energy Formula (6).

(6) $$\Delta G=-RT\ln K_{d}$$

The *ΔS* and *ΔH* at different temperatures can be calculated using the Van’t Hoff equation, with the calculation formulas shown in Equations (7) and (8).

(7) $$\ln K_{d}=\Delta S/R-\Delta H/\left(RT\right)$$

(8) $$K_{d}=q_{e}/C_{e}$$

All experiments were performed in triplicates, and the average values were calculated. The amount of adsorbed biochar was calculated using Equation (9).

(9) $$q_{e}=\left(C_{0}-C_{e}\right)V/M$$

Since *K_d_* = *q_e_*/*C_e_* has the dimension of L/g, it was converted into the dimensionless standard equilibrium constant *K_d_^⊖^* = *K_d_ (C^⊖^/q^⊖^)* to satisfy the dimensional requirement for rigorous thermodynamic calculation, where *C^⊖^* = 1 mg/L and *q*^*⊖*^ = 1 mg/g. The thermodynamic parameters calculated from this dimensionless standard equilibrium constant correspond to standard-state thermodynamic quantities.

Where *qe* denotes the adsorption amount of biochar at adsorption equilibrium (mg/g), *C*_0_ is the initial concentration (mg/L), *C_e_* is the ammonium nitrogen concentration at equilibrium (mg/L), *V* represents the solution volume, and M denotes the amount of biochar added.

#### 2.4.4. Description of the Experimental Method for the Combined Effect of Modified Biochar

To evaluate the ammonium nitrogen removal performance of EM-modified biochar, batch experiments were conducted under the conditions specified in Section 2.4.1, Section 2.4.2 and Section 2.4.3: 1.0 g of modified biochar was weighed, added to 50 mL of ammonium nitrogen solution, and shaken at 200 rpm and the designated temperature, and samples were filtered through a 0.45 μm membrane to measure the residual ammonium nitrogen concentration.

To distinguish the contributions of adsorption and biodegradation, two control experiments were set up in this study:

(1) Sterilized modified biochar control: EM-modified biochar was sterilized in an autoclave at 121 °C for 30 min to inactivate the immobilized microorganisms, while other experimental conditions were the same as for the modified biochar. The ammonium nitrogen removal in this group represents the contribution from purely physicochemical adsorption.

(2) EM culture control: The same volume of EM culture used in the modification process (about 1.67 mL) was collected, washed by centrifugation (8000 rpm, 10 min), and resuspended in the same volume of deionized water, then directly mixed with 1.0 g of original biochar without the 48 h dark incubation and 40 °C drying steps. Other experimental conditions were the same as for the modified biochar. This group was used to verify whether the immobilization process itself provides any enhancement beyond simple physical mixing.

### 2.5. Material Characterization and Analysis Methods

The morphological characteristics of the biochar were observed using scanning electron microscopy (SEM, SIGMA500, Carl Zeiss, Oberkochen, Germany). The specific surface area and pore structure of the biochar were analyzed using a specific surface area and porosity analyzer (Autosorb iQ, Quantachrome Instruments, Boynton Beach, FL, USA). The infrared spectra of the biochars were measured using a Fourier-transform infrared spectrometer (FT-NIR, Antaris II, Thermo Fisher Scientific, Waltham, MA, USA). The concentration of ammonium nitrogen in the solution was measured using an ultraviolet-visible spectrophotometer (Lambda series, PerkinElmer, Shelton, CT, USA). Metagenomic sequencing was performed using the DNBSEQ-T7 platform (MGI Tech Co., Ltd., Shenzhen, China).

## 3. Results and Discussion

### 3.1. Molecular Mechanisms and Degradation Characteristics of Ammonium Nitrogen in EM Bacteria

#### 3.1.1. Metagenomic Analysis of EM Bacteria

**Figure 2:**
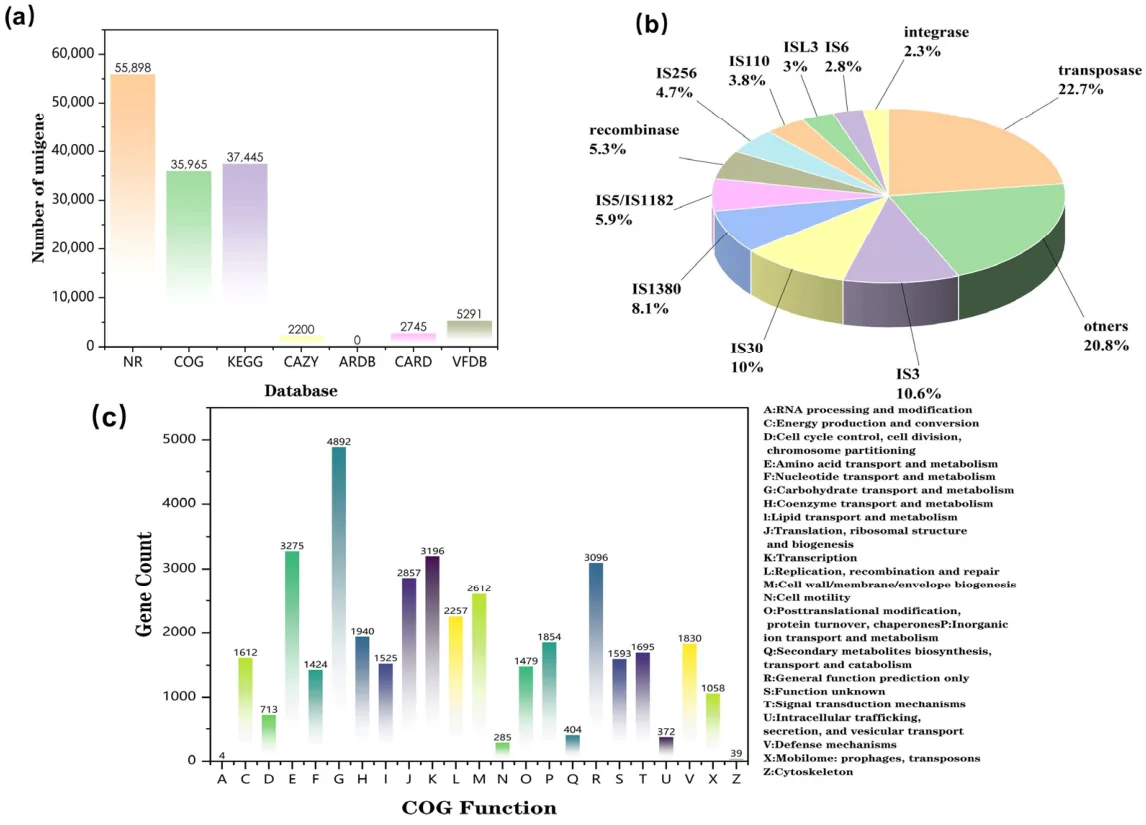
Genomic annotation and functional classification of the strain. (**a**) Functional annotation of unigenes. (**b**) Gene count distribution. (**c**) COG functional classification.

Strain A1 was isolated from a commercial EM microbial solution via serial dilution and plate screening and was selected as the representative isolate owing to its superior ammonium removal performance. Although the commercial EM consortium consists of diverse microbial species, strain A1 was subjected to genomic analysis to clarify the genetic basis of HN-AD metabolic potential within the EM-derived microbial community. NR database annotation showed that this strain was Bacillus thuringiensis, with more than 99% consistency with the ammonia-nitrogen-degrading strain EM-A1 (MW551532.1) [21]. KEGG functional annotation (Figure 2a) showed that 22,000 genes in the pathways were involved in metabolism, covering core pathways such as nitrogen, energy, and carbohydrate metabolism [29]. The COG functional classification (Figure 2c) further revealed the metabolic characteristics of the strains: carbohydrate transport and metabolism (Category G, 4892 genes) was the most abundant, indicating that the strain possesses a high efficiency in carbon source utilization [29]; amino acid transport and metabolism (Class E, 3275 genes) came next, reflecting its strong nitrogen assimilation capacity; and energy production and conversion (Category C, 1612 genes) were also relatively abundant, providing a continuous supply of ATP for the ammonium nitrogen degradation process. These functional characteristics collectively form the metabolic foundation for efficient nitrogen removal by this strain. Key enzyme genes involved in the ammonium nitrogen degradation pathway identified from the genome are summarized in Table 1. The *amoA* gene (K10944, read count 980) encodes ammonia monooxygenase, which catalyzes the initial oxidation of ammonia (NH_3_) to hydroxylamine NH_2_OH). The *hao* gene (K10535, read count 1134) encodes hydroxylamine oxidoreductase, which catalyzes the conversion of hydroxylamine to nitrite (NO_2_^−^), serving as a critical step to mitigate hydroxylamine toxicity. The *napA* gene (K02567, read count 1223) encodes periplasmic nitrate reductase, which is responsible for reducing nitrate (NO_3_^−^) to nitrite (NO_2_^−^). The *nirK* gene (K00368, read count 1440) encodes copper-containing nitrite reductase and catalyzes the reduction of nitrite to nitric oxide (NO). The *norB* gene (K04561, read count 1470) encodes nitric oxide reductase, which catalyzes the conversion of NO to nitrous oxide (N_2_O) for timely removal of highly toxic intermediates. Finally, the *nosZ* gene (K00376, read count 1432) encodes nitrous oxide reductase, which mediates the final step to reduce N_2_O to nitrogen gas (N_2_).

The synergistic presence of these six key functional genes, namely *amoA*, *hao*, *napA*, *nirK*, *norB*, and *nosZ*, covers the complete heterotrophic nitrification–aerobic denitrification (HN-AD) pathway from NH_3_ to N_2_. The read counts of these genes ranged from 980 to 1470 at comparable levels, indicating their stable existence and sufficient sequence coverage within the assembled genome. Nevertheless, it should be emphasized that these read counts only represent DNA sequence coverage instead of transcriptional activity (mRNA abundance) or protein expression. The enzyme activities of ammonia monooxygenase (AMO, 0.37 U/mg) and hydroxylamine oxidoreductase (HAO, 0.88 U/mg) further verified the functional expression of the initial nitrification steps. Collectively, these genomic and enzymatic data suggest that strain A1 possesses the genetic capacity for ammonium nitrogen transformation via the HN-AD pathway. Nevertheless, gene presence does not by itself confirm the actual metabolic flux through this pathway under the experimental conditions [30]. CARD annotation identified ABC efflux pump genes *patA* and *patB* [31,32] in the genome of strain A1, with read coverage of 79,034 and 22,704, respectively. Combined with the detection of mobile genetic elements (transposases and insertion sequences), these findings indicate that strain A1 harbors the genetic repertoire for environmental stress tolerance, including efflux-mediated detoxification and genomic plasticity. Nevertheless, the mere presence of these genes cannot confirm their transcriptional expression or functional activity under the experimental conditions of this study. Further investigations using transcriptomic analysis (RNA-seq) together with functional validation approaches (e.g., gene knockout and enzyme activity assays) are needed to clarify their actual roles in the environmental tolerance of strain A1.

Based on the presence of the hao and norB genes and the absence of anammox-related genes (e.g., hzs), the ammonium removal pathway of strain A1 can be classified as heterotrophic nitrification–aerobic denitrification (HN-AD) [33]. This pathway allows the strain to convert ammonium to hydroxylamine (via AMO), nitrite (via HAO), and gaseous nitrogen oxides (via NorB) under aerobic conditions without requiring separate anoxic/anaerobic stages. The above genomic features provide a molecular basis for interpreting the macroscopic ammonium degradation performance of EM bacteria under diverse environmental conditions. Specifically, the presence of intact heterotrophic nitrification–aerobic denitrification (HN-AD) genes including hao and norB indicates that the strain possesses enzymatic capacity for both nitrification and denitrification, which endows it with efficient ammonium removal over a wide range of temperature and pH values. The high abundance of ABC efflux pump genes patA and patB implies inherent tolerance toward toxic intermediates such as hydroxylamine, as well as external environmental stressors, which contributes to its broad pH adaptability. In addition, mobile genetic elements (transposases and insertion sequences) reflect genomic plasticity, allowing the strain to sustain stable metabolic activity under fluctuating conditions such as variable temperature.

#### 3.1.2. The Impact of Environmental Factors on the Degradation Efficiency of EM Bacteria

Temperature, pH, and initial ammonium concentration significantly regulated the degradation efficiency of the EM bacteria.

**Figure 3:**
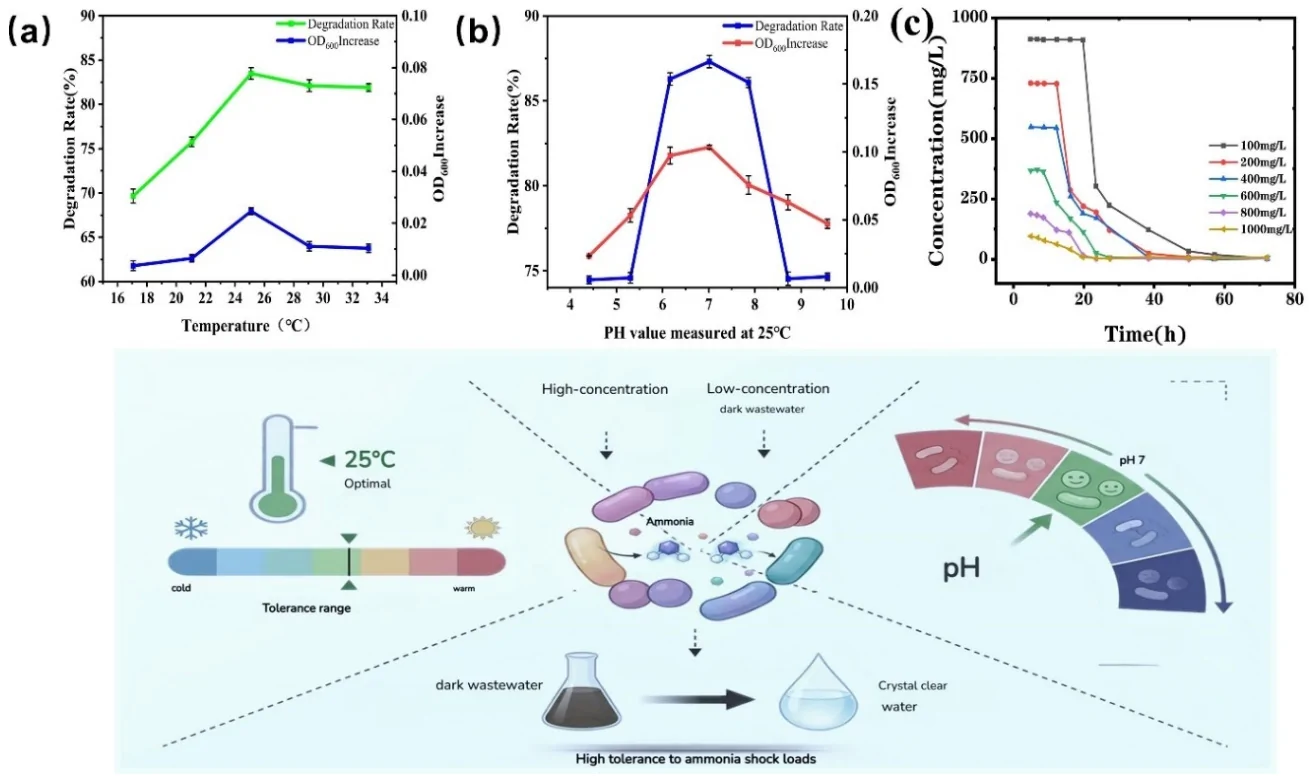
Effects of environmental factors on the ammonium degradation efficiency of EM bacteria. (**a**) Effect of temperature on degradation efficiency. (**b**) Effect of pH on degradation efficiency. (**c**) Effect of initial ammonium concentration on degradation efficiency.

Temperature (Figure 3a): The degradation rate first increased and then decreased with temperature, peaking at 83.5% at 25 °C and remaining above 70% at 15 °C and 35 °C. This is attributed to enzymatic reaction kinetics: low temperature inhibits enzyme activity, whereas high temperatures denature enzymes [34]. The temperature of 25 °C fell within the optimal range for key enzymes (AMO and HAO). EM bacteria maintain high efficiency across 15–35 °C and demonstrate strong adaptability at these temperatures. Mobile genetic elements (transposases, IS sequences) confer genetic plasticity, enabling the strain to sustain denitrification function under fluctuating temperatures through gene recombination and horizontal gene transfer.

pH (Figure 3b): The degradation rate was highest at pH 7 (82.35%) and severely inhibited at pH 4 and 10. Mechanistically, ammonia exists as NH_4_^+^ and NH_3_ (pKa ≈ 9.25). Under acidic conditions, NH_4_^+^ predominates; however, excess H+ disrupts proton gradients and ATP synthesis. Under alkaline conditions, NH_3_ increases, penetrates the membrane, and accumulates intracellularly, causing toxicity [35]. EM bacteria perform optimally at neutral to slightly alkaline pH and benefit from the combined regulation of membrane permeability and ammonia transport.

Initial ammonium concentration (Figure 3c): Low concentration (100 mg/L) was completely degraded within 8 h. At a high concentration (1000 mg/L), the initial degradation rate was inhibited, but complete removal was ultimately achieved. This reveals a substrate adaptation mechanism: at low concentrations, ammonia serves as a nitrogen and energy source matching metabolic demand; at high concentrations, the accumulation of ammonia and intermediates (e.g., hydroxylamine) causes toxicity [36]. EM bacteria adapt by (i) inducing AMO and HAO expression to increase metabolic flux, (ii) using ABC efflux pumps to expel toxic intermediates, providing instant tolerance to 500–1000 mg/L ammonia shocks, and (iii) redirecting more carbon sources to energy metabolism to support high loads of ammonia. The growth curve (OD_600_) paralleled degradation, confirming substrate-dependent proliferation.

These molecular features form the phenotype–molecular evidence chain of strain A1. The efflux pump system provides instant tolerance to peak loads, while mobile genetic elements confer long-term adaptability, together ensuring stable operation of EM bacteria in various engineering scenarios—adapting to diurnal temperature fluctuations (15–35 °C) and low nutrient conditions in ecological ditches or constructed wetlands and tolerating intermittent flow and pollutant fluctuations in farmland drainage purification.

The genomic traits characterized in Section 3.1.1 deliver mechanistic molecular interpretations for the macroscopic ammonium degradation performance described above. The complete set of heterotrophic nitrification–aerobic denitrification (HN-AD) genes hao and norB underpin the strain’s high degrading efficiency across 15–35 °C and its strong tolerance against acute ammonium shock loads up to 1000 mg/L. The presence of ABC efflux pump genes (*patA*/*patB*) in the genome of strain A1 indicates a potential genetic basis for tolerating toxic intermediates (e.g., hydroxylamine) and external environmental stressors, which may account for the broad-pH adaptability of strain A1. Nevertheless, direct evidence confirming that these genes exert efflux function or mediate stress tolerance under the experimental conditions is lacking in the present study.

### 3.2. Structural Evolution and Adsorption Performance of Biochar

#### 3.2.1. Morphology and Pore Structure Characteristics of Biochar

**Figure 4:**
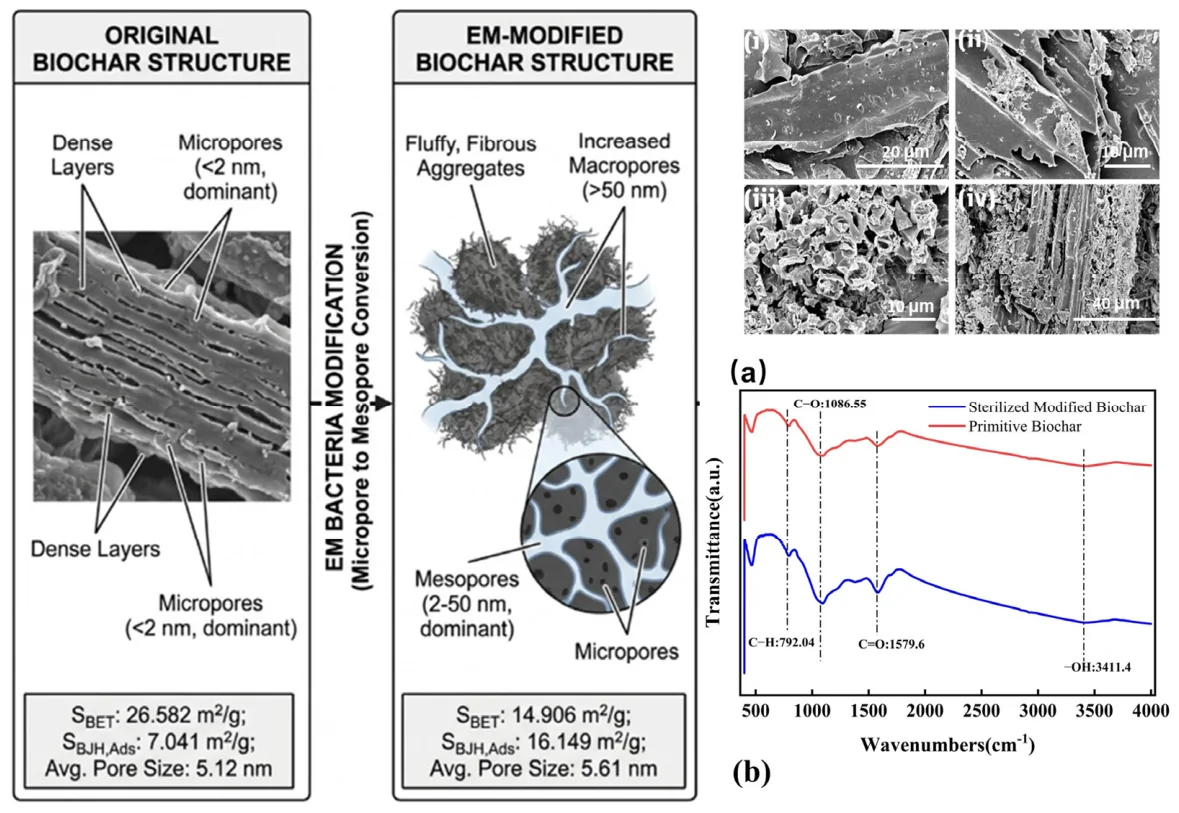
Morphological characteristics and pore structures of pristine and modified biochars. (**a**) SEM images of pristine and modified biochars. (**b**) FTIR spectra of the primitive and modified biochars.

In terms of micro-morphology, Figure 4a illustrates that pristine biochar presents compact lamellar structures (Figure 4a(i,ii)), while EM-modified sterilized biochar transforms into loose fibrous aggregates (Figure 4a(iii,iv)) with continuous large pore channels. All biochar samples underwent standard high-temperature pretreatment prior to SEM observation, eliminating the interference of thermal treatment differences in microscopic morphology. The pore structural parameters of pristine and EM-modified sterilized biochar are summarized. After EM immobilization modification, the total BET specific surface area S_BET_ declined from 26.582 m^2^/g to 14.906 m^2^, whereas the BJH mesoporous adsorption area S_BJH,Ads_ rose sharply from 7.041 m^2^/g to 16.149 m^2^ (a 129.36% increment), and the average pore diameter increased from 5.1243 nm to 5.6144 nm (9.56% larger). These changes indicate a redistribution of pore-size distribution toward the mesopore range, with an increased proportion of mesopores and a corresponding decrease in microporosity. The observed decrease in total BET surface area is likely attributable to pore blockage or occupancy by microbial cells and their metabolic products, rather than the physical collapse of the pore structure. Such structural evolution may be associated with microbial colonization and metabolic activity during the modification process.

Since all test samples received identical high-temperature pretreatment before SEM characterization, the distinct morphological differences between pristine and modified biochar are primarily attributable to the EM modification process (including microbial colonization and associated physicochemical interactions), rather than autoclave sterilization. Comparisons of pore size distribution and FTIR spectra between pristine biochar and sterilized EM-modified biochar revealed no substantial differences, preliminarily suggesting that the bulk structure of biochar was not drastically altered under the present sterilization conditions. Nevertheless, this comparison cannot completely rule out subtle changes in surface chemical properties induced by moist heat sterilization, including variations in mineral phases, surface charge, and leachable organic matter. Furthermore, routine sample pretreatment for SEM and FTIR analysis (e.g., vacuum drying and KBr pressing) inevitably inactivates microorganisms, making direct paired characterization of the identical viable composite before and after autoclaving unfeasible. Therefore, the present structural and spectral results serve only as auxiliary reference and cannot provide definitive proof that autoclaving exerts no influence on biochar properties.

Published literature has demonstrated that hierarchical micropore–mesopore frameworks accelerate solute diffusion and maximize the utilization of adsorption active sites [37]. Mesopore-dominated architecture brings two practical merits for water remediation: under continuous flow conditions in constructed wetlands or ecological ditches, widened pore channels cut down hydrodynamic resistance and mitigate filler clogging, while sufficient specific surface area is retained to guarantee ammonium adsorption capacity, realizing simultaneous fast mass transfer and high removal efficiency.

FTIR spectra (Figure 4b) show negligible shifts in peak position, intensity, and shape between pristine and EM-modified sterilized biochar, indicating that EM colonization hardly alters the categories and distribution of surface functional groups, consistent with previous research conclusions [38]. Two underlying reasons account for this phenomenon: (i) EM strains only colonize pore interiors and utilize carbon substrates without destroying the aromatic skeleton of biochar; (ii) trace metabolic by-products fail to generate detectable new functional groups due to concentrations below the instrument detection limit.

Combined with intra-particle diffusion kinetic data in Table 2, the increased mesopore surface area and enlarged average pore diameter correspond to a prominent rise of *K_id_* from 0.532 to 0.953, suggesting that the shifted pore-size distribution reduces intragranular diffusion resistance and accelerates ammonium mass transfer. On the basis of the above structural optimization characteristics, adsorption kinetic tests were further conducted to reveal the rate-controlling mechanism of ammonium uptake on modified biochar.

#### 3.2.2. Analysis of Adsorption Kinetic Behavior

Three kinetic models (pseudo-first-order, pseudo-second-order, and intra-particle diffusion) were adopted to fit ammonium adsorption data of four biochar groups, and all fitting parameters are listed in Table 2. The correlation coefficients (*R*^2^) of the pseudo-second-order model for all samples exceeded 0.987. The equilibrium adsorption capacities with standard deviations from triplicate tests were as follows: pristine biochar, 0.613 ± 0.021 mg/g; physical mixture of pristine biochar and free EM bacteria, 0.692 ± 0.018 mg/g; sterilized EM-modified biochar, 1.212 ± 0.022 mg/g; and unsterilized viable EM-modified biochar, 1.322 ± 0.017 mg/g.

**Figure 5:**
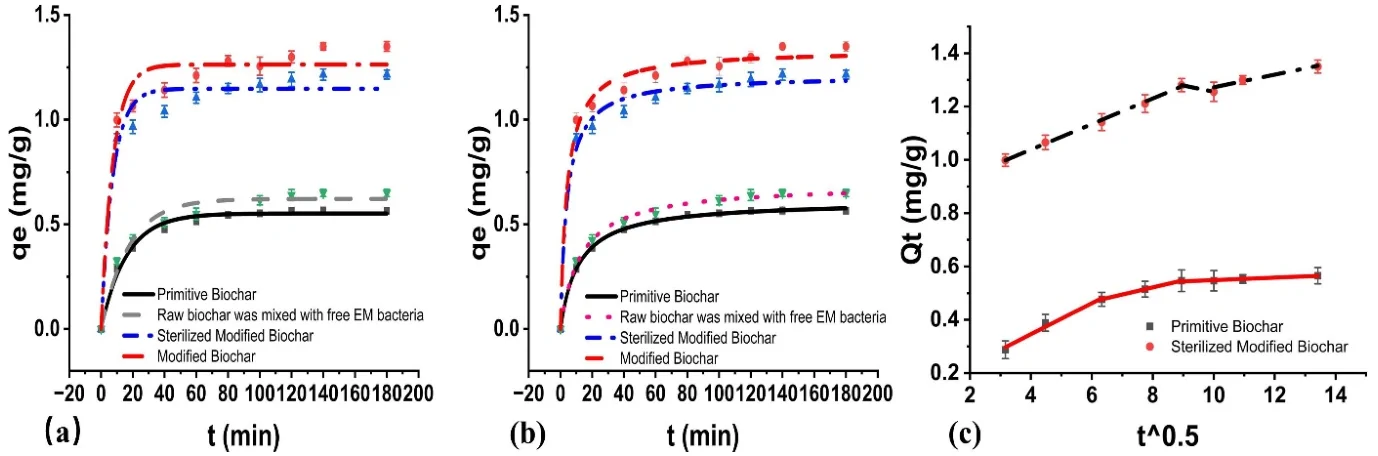
Fitting parameters of pseudo-first-order, pseudo-second-order, and intra-particle diffusion kinetic models for ammonium adsorption on four types of biochar. (**a**) Pseudo-first-order kinetic model fitting parameters. (**b**) Pseudo-second-order kinetic model fitting parameters. (**c**) Intra-particle diffusion model fitting parameters.

Adsorption data of biochar were fitted by pseudo-first-order, pseudo-second-order and intra-particle diffusion models, as illustrated in Figure 5.

A two-tailed independent sample *t*-test was performed for the pristine biochar group and the physical mixture group (*t* = 6.02, *d_f_* = 4, *p* < 0.05) [39]. The results showed that simple mixing slightly improved adsorption performance compared with pristine biochar, while the capacity increment was only 0.079 mg/g. Free bacteria were only loosely attached to the outer surface of biochar without pore protection. They tended to detach under hydraulic shear force and were sensitive to environmental stress, making it impossible to construct a long-term stable synergistic adsorption–biodegradation system.

The capacity difference between sterilized and unsterilized EM-modified biochar was used to quantify the contribution of microbial degradation. Only physicochemical adsorption occurred in sterilized samples, with an equilibrium capacity of 1.212 ± 0.022 mg/g; the unsterilized composite possessed both adsorption and biodegradation capacities, reaching 1.322 ± 0.017 mg/g. The two-tailed *t*-test yielded *t* = −8.12, *d_f_* = 4, *p* < 0.05, indicating that the difference between the two groups was statistically significant. The capacity difference between sterilized and unsterilized EM-modified biochar was 0.110 mg/g. The difference in capacity between sterilized and viable EM-modified biochar was 0.110 mg/g, indicating that microbial activity drove ammonium transformation in addition to physicochemical adsorption.

It is important to note that this 0.110 mg/g difference should be interpreted with caution. Our experimental design only comprised two groups (sterilized versus viable EM-modified biochar) and did not include a control of autoclaved pristine biochar. Accordingly, the observed difference cannot definitively disentangle microbial biodegradation effects from potential physicochemical modifications to the biochar matrix caused by autoclaving. More rigorous quantification would require a third control group (autoclaved pristine biochar) to separate these two sets of effects.

This result suggests that 48 h static dark incubation facilitated stable colonization of EM bacteria inside mesopores. Protected by pore channels, bacteria sustained degradation activity and provided considerable extra nitrogen removal capacity, verifying that intra-pore immobilization is the core prerequisite to realize synergistic adsorption–biodegradation.

The intra-particle diffusion constant (*K_id_*) was used to quantify the mass transfer resistance of ammonium inside pores. Two pure adsorption systems without microbial interference (pristine biochar and sterilized modified biochar) were selected to establish the quantitative relationship between pore structure and mass transfer performance. Pristine biochar was dominated by narrow micropores with a low *K_id_* value of 0.53. After EM modification, the pore-size distribution shifts toward the mesopore range; the mesoporous specific surface area increased by 129.4%, the average pore size expanded by 9.6%, and *K_id_* rose significantly to 0.95, representing a 79.2% improvement in mass transfer efficiency. A clear positive correlation was observed between mesopore proportion, average pore size, and *K_id_*, confirming that directional pore expansion effectively alleviated intra-particle diffusion limitation. The widened continuous pore channels reduced diffusion barriers and accelerated the migration of ammonium ions toward deep internal adsorption sites, which microscopically explained the remarkable increase in saturated adsorption capacity after modification.

#### 3.2.3. Analysis of Isothermal Adsorption Behavior

**Figure 6:**
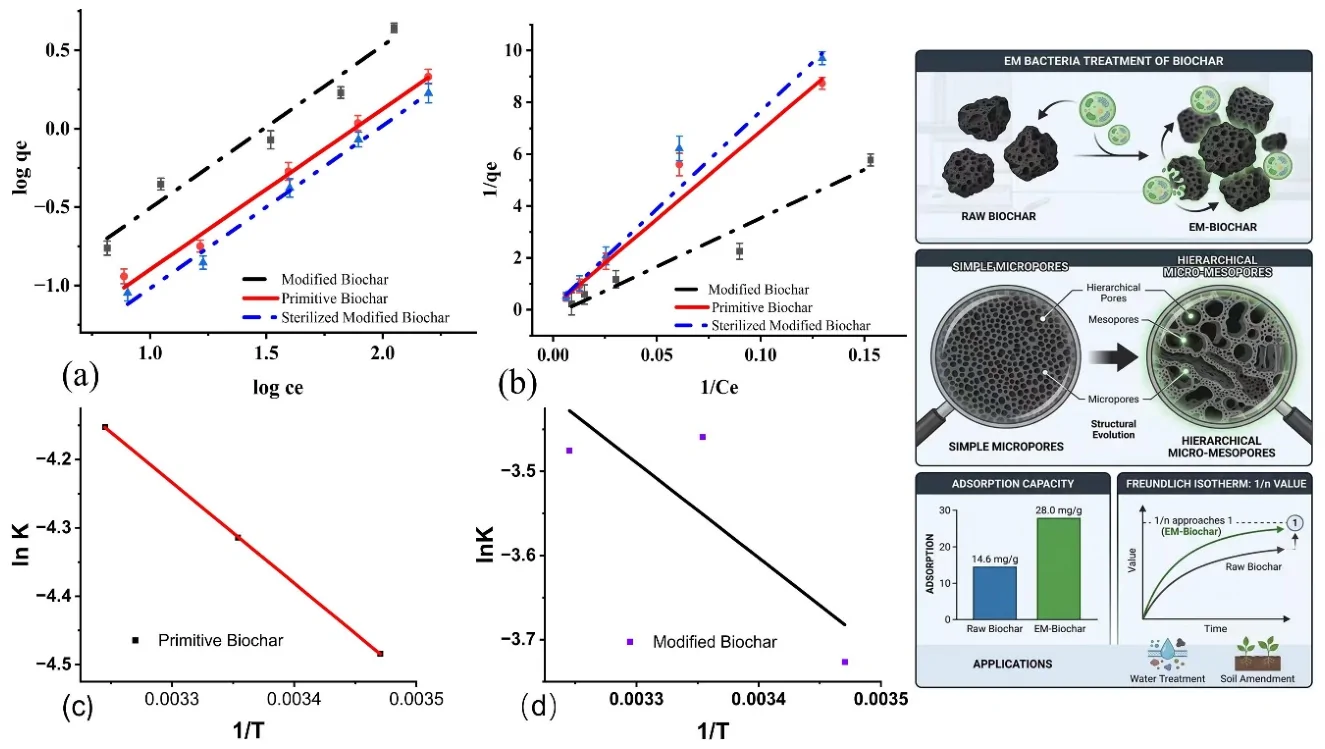
Isothermal adsorption of biochar before and after modification, thermodynamic data diagram, and mechanism diagram. (**a**) Freundlich adsorption isotherms of ammonium nitrogen on pristine and modified biochar. (**b**) Langmuir adsorption isotherms of ammonium nitrogen on pristine and modified biochar. (**c**,**d**) Van’t Hoff plots of pristine biochar and sterilized modified biochar.

Two classic isotherm models, Langmuir (monolayer homogeneous adsorption) and Freundlich (heterogeneous multilayer adsorption), were adopted to fit the adsorption data of pristine biochar and sterilized EM-modified biochar (Figure 6a,b). Since immobilized microorganisms in the unsterilized viable composite continuously consume ammonium in the liquid phase, and true adsorption equilibrium cannot be achieved, only the two pure adsorption systems were selected for quantitative analysis. The fitting results of unsterilized modified biochar were used for auxiliary reference only and excluded from comparative evaluation. All parameters listed in Table 3 correspond to these two equilibrium adsorption systems.

The maximum adsorption capacity (*q_max_*) of pristine biochar fitted by the Langmuir model was 0.73 mg/g with a correlation coefficient (*R*^2^) of 0.956, while the *R*^2^ of the Freundlich model was 0.985. For sterilized EM-modified biochar, the Langmuir *q_max_* reached 1.215 mg/g (*R*^2^ = 0.946), increasing by 66.4% compared with the pristine material, and the Freundlich *R*^2^ was 0.959. The similar *R*^2^ values of the two models suggest that monolayer adsorption and heterogeneous multilayer adsorption may coexist on the biochar surface. Therefore, no single mechanism was concluded. Instead, ammonium adsorption is jointly governed by surface heterogeneity and monolayer chemisorption, in which chemisorption serves as the rate-limiting step, as inferred from the kinetic analysis (Section 3.2.2).

The Langmuir affinity constant *K_L_* was 0.139 L/mg for pristine biochar and 15.7 L/mg for sterilized modified biochar. The approximately 113-fold increase in *K_L_* after modification reflects enhanced accessibility of adsorption sites originating from optimized pore structure (129.4% increase in mesoporous specific surface area and 9.6% expansion of average pore size). This value is consistent with published data of other chemically or biologically modified biochars. The separation factor *R_L_* = 1/(1 + *K_L_* C0) fell within the favorable range (0 < R_L_ < 1), further supporting the above conclusion.

The Freundlich index 1/n was 1.023 for pristine biochar and 1.037 for sterilized modified biochar. Both values were slightly higher than 1, indicating superlinear growth of adsorption capacity with equilibrium concentration within the tested range (10–200 mg/L), which may be attributed to surface heterogeneity or adsorbate–adsorbate interactions under high loading. Notably, 1/n > 1 does not imply poor adsorption performance; on the contrary, it demonstrates that the adsorbent maintains efficient removal capacity under fluctuating pollutant loads, which is desirable for agricultural runoff treatment. Moreover, a 1/n value close to 1 only indicates near-linear adsorption behavior within the investigated concentration window and cannot independently validate either the Langmuir or Freundlich model.

Combined with SEM and BJH pore characterization, EM modification shifted the pore-size distribution toward the mesopore region and increased both the mesoporous specific surface area and average pore size. These changes lowered intra-particle diffusion resistance and raised the intra-particle diffusion constant *K_id_* from 0.532 to 0.953. Optimized mass transfer conditions together with sufficient monolayer adsorption sites markedly improved the saturated adsorption capacity. The isotherm fitting results were highly consistent with microstructural characteristics.

#### 3.2.4. Analysis of Adsorption Thermodynamic Behavior

Viable EM-modified biochar cannot reach stable adsorption equilibrium due to continuous biodegradation of ammonium by immobilized strains. Therefore, only pristine biochar and sterilized EM-modified biochar (two pure adsorption systems) were selected for thermodynamic calculation, and all thermodynamic parameters are listed in Table 3 and Figure 6c,d.

At 298.15 K, the Gibbs free energy *ΔG^⊖^* of pristine biochar was 10.693 kJ/mol, while sterilized EM-modified biochar possessed a *ΔG^⊖^* value of 8.574 kJ/mol. The positive *ΔG^⊖^* values of both groups indicated that ammonium adsorption was a non-spontaneous process at ambient temperature; the obvious decline of *ΔG^⊖^* after modification suggested that pore structural optimization by EM bacteria greatly improved the spontaneity of adsorption.

The enthalpy change *ΔH^⊖^* of all samples was positive (pristine biochar: 12.240 kJ/mol; sterilized modified biochar: 9.393 kJ/mol), suggesting that ammonium uptake was an endothermic reaction, and higher temperature facilitated the adsorption process. The reduced *ΔH^⊖^* of modified biochar implied lower energy demand for ammonium adsorption, and the adverse effect of temperature fluctuation on adsorption capacity was alleviated.

The entropy changes *ΔS^⊖^* of pristine biochar and sterilized modified biochar were 5.195 J·mol^−1^·K^−1^ and 1.986 J·mol^−1^·K^−1^, respectively. Positive *ΔS^⊖^* values revealed increased disorder at solid-liquid interface during adsorption. Massive mesopores were generated after EM modification, forming regular and continuous pore channels. Ammonium molecules were arranged more orderly inside pores after adsorption, resulting in a remarkably reduced entropy increment of the whole system.

Combined with the intra-particle diffusion kinetic results above, EM modification induced a shift of pore-size distribution toward the mesopore range with elevated mesoporous specific surface area, which widened mass-transfer channels and lowered the energy barrier of adsorption (reflected by simultaneous decline of *ΔG^⊖^* and *ΔH^⊖^*), which macroscopically contributed to the prominent growth of Langmuir saturated adsorption capacity. The variation trends of thermodynamic parameters were highly consistent with pore structure and mass transfer characteristics, illustrating the regulatory mechanism of pore optimization on ammonium adsorption from the perspective of energy change.

The structural, kinetic, isothermal, and thermodynamic datasets are mutually consistent, collectively outlining a complete sequential pathway. EM modification shifts the pore-size distribution toward the mesopore range, accompanied by a 129.4% increase in mesoporous specific surface area and a 9.6% enlargement in average pore size. The widened pore channels effectively mitigate intragranular mass transfer resistance and accelerate internal diffusion, as evidenced by a 79.1% elevation in *K_id_*. Improved mass transfer facilitates greater accessibility to inner monolayer adsorption sites, which is reflected by a 66.4% enhancement in the Langmuir maximum adsorption capacity *q_m_*. Ultimately, the declines in *ΔG^⊖^* (approximately 2 kJ/mol reduction) and *ΔH^⊖^* (from 12.24 to 9.39 kJ/mol) demonstrate that the above structural and kinetic optimizations jointly lower the adsorption energy barrier. This cascade mechanism—pore expansion → accelerated mass transfer → enhanced accessibility of adsorption sites → reduced adsorption energy barrier—provides a coherent mechanistic explanation for the boosted ammonium adsorption capacity of EM-modified biochar.

## 4. Discussion

Agricultural non-point source pollution, primarily driven by nitrogen loss from farmland drainage and livestock wastewater, seriously impedes sustainable agricultural development and ecological improvement in China. Straw-derived biochar realizes the recycling of agricultural waste and can intercept nitrogen loss within agroecosystems. Therefore, understanding the ammonium nitrogen removal mechanism of biochar–microbe composites is critical for advancing precise nitrogen-management strategies for farmland. In the present work, sterilization-control experiments were used to distinguish physicochemical adsorption and microbial biodegradation effects. The results indicate that physicochemical adsorption constitutes the dominant ammonium nitrogen removal pathway, whereas microbial biodegradation delivers an auxiliary removal effect. Although the relative contribution from microbial processes is comparatively low, microbial activity may endow the composite with sustainable nitrogen-transformation capacity that conventional adsorbents lack, which could support long-term nitrogen interception performance. Molecular biological analysis suggests that the isolated strain A1, identified as *Bacillus thuringiensis*, shares more than 99% sequence homology with previously reported efficient nitrogen-transforming bacterial strains. The genome of strain A1 harbors a full set of functional genes (*amoA*, *hao*, *napA*, *nirK*, *norB*, *nosZ*) associated with the heterotrophic nitrification–aerobic denitrification (HN-AD) pathway, alongside ABC efflux-pump genes (*patA*/*patB*) and mobile genetic elements. These findings point to the genetic potential of strain A1 for nitrogen transformation and stress resistance, yet gene detection based on genomic read counts cannot prove transcriptional expression or real-world metabolic flux under the tested conditions. High gene read counts coincided with optimal degradation performance at 25 °C and neutral pH, which is consistent with the occurrence of HN-AD-related metabolic activity but does not serve as definitive evidence.

The improved ammonium-removal performance of the composite is largely ascribed to pore-structure modification caused by microbial immobilization. EM treatment shifted the pore-size distribution toward the mesopore region; the mesopore-specific surface area increased by 129.4%, and the average pore size rose by 9.6%. Such optimized pore architecture reduces intraparticle mass-transfer resistance and raises the intraparticle diffusion coefficient by 79%, which activates more monolayer adsorption sites and yields a 66.4% increase in the Langmuir maximum adsorption capacity. Reduced *ΔG^⊖^* and *ΔH^⊖^* imply a decreased adsorption energy barrier and weaker temperature dependence after modification. By contrast, simple physical mixing of raw biochar and free EM bacteria hardly improved ammonium removal, which suggests that in-pore immobilization of microorganisms is a prerequisite for obtaining combined adsorption–biodegradation effects. The EM-modified biochar achieved an ammonium adsorption capacity of 1.322 mg/g, nearly twice the value for pristine biochar (0.613 mg/g). This property facilitates rapid ammonium capture during rainfall-runoff events, and enhanced mass-transfer may support nitrogen retention under dynamic-flow conditions in agricultural ditches and constructed wetlands. Compared with H_2_O_2_-modified rice-husk biochar and KMnO_4_-modified wheat-straw biochar reported in published literature, the EM-modified corn-straw biochar prepared in this study shows competitive adsorption capacity under relatively mild modification conditions [8,9].

Collectively, pore-structure optimization underpins dominant physicochemical adsorption, while pore-immobilized microorganisms provide auxiliary biodegradation, jointly forming the proposed synergistic ammonium-removal mechanism of the composite. In this study, only pristine biochar and sterilized EM-modified biochar were compared, while the control of autoclaved pristine biochar was not included. Therefore, although no prominent differences were observed between the two tested groups, we cannot rule out subtle physicochemical changes induced by moist-heat autoclaving, including mineral phase transformation, surface charge shift, and release of leachable organic matter. Direct paired characterization of identical viable composites before and after autoclaving remains experimentally unfeasible owing to unavoidable microorganism inactivation during routine SEM and FTIR sample pretreatment. Kinetic fitting implies that chemisorption may act as the rate-limiting step, reflecting relatively homogeneous adsorption sites and stable adsorption behavior. Unlike conventional adsorbents that merely retain nutrients temporarily, the EM-modified composite combines fast ammonium adsorption with potential biological transformation. Adsorbed ammonium may be transformed by immobilized microorganisms or slowly released for crop uptake, which may improve nitrogen-use efficiency and lower chemical-fertilizer inputs. Benefiting from nutrient-capturing and potential biological-regulation functions, the composite exhibits application prospects in ecological-ditch purification, constructed-wetland remediation, soil amelioration, and irrigation-water treatment. Moreover, the composite is produced from waste corn straw and commercial EM inoculant, which provides advantages of low cost, good environmental compatibility, and abundant raw-material supply and conforms to the requirements for agricultural-waste recycling and green agricultural-pollution mitigation.

Several important limitations should be acknowledged when interpreting the present results. All experiments were performed in deionized-water laboratory systems, ignoring interference from coexisting substances in real agricultural wastewater. Cations such as Ca^2+^ and Mg^2+^ can compete for adsorption sites and may suppress microbial metabolism; when cation concentrations exceed 50 mg/L, ammonium-adsorption capacity can decrease by 15–30%. Dissolved organic matter widespread in farmland drainage may block mesopores, cover active sites, and hinder substrate and oxygen transport for immobilized microbes, which may impair the long-term stability of the apparent microbial contribution observed in this study. In addition, the deionized-water setup lacks the HCO_3_^−^/CO_3_^2−^ buffering capacity of natural agricultural water and cannot reproduce field pH fluctuations ranging from 6.0 to 8.5, which may alter NH_4_^+^/NH_3_ speciation and the surface electrostatic properties of modified biochar.

With respect to quantifying adsorption–biodegradation partitioning, the autoclaving approach adopted in this study has intrinsic drawbacks. First, 121 °C autoclaving may change mineral phases, surface-charge characteristics, or leachable organic fractions of biochar, and side-by-side characterization of identical intact composites before and after sterilization was not conducted. Second, sample pretreatment for SEM and FTIR (vacuum drying, KBr pellet pressing) inevitably inactivates living microorganisms, so direct characterization of viable composite materials remains experimentally unfeasible. Accordingly, the apparent adsorption and microbial contributions inferred from sterilization-control tests represent apparent values valid only for the given experimental conditions rather than absolute quantitative constants. The apparent microbial effect may include both assimilatory nitrogen incorporation into microbial biomass and dissimilatory HN-AD-type transformation; owing to the absence of complete nitrogen-balance monitoring and quantification of intermediate and gaseous nitrogen products, the relative magnitude of these two processes cannot be distinguished in the current dataset. Chemical-inhibition treatments (e.g., 0.2% sodium azide) are recommended in future work to achieve more rigorous separation of adsorption and biodegradation.

It should also be noted that commercial EM inoculant is a complex microbial consortium. Strain A1 used for genomic analysis was isolated as one representative functional strain from the original EM community. However, the actual community composition colonized on EM-modified biochar was not characterized in this study. Therefore, although HN-AD-related functional genes were detected in strain A1, we cannot confirm that strain A1 dominates the biofilm community or independently accounts for the observed apparent biodegradation contribution. Subsequent research could employ 16S rRNA amplicon sequencing and strain-specific qPCR to unravel community succession and identify key functional populations within biochar-microbe composite systems. Metagenomic data reveal the genetic potential of strain A1 for nitrogen transformation and stress tolerance, yet DNA-level gene detection does not guarantee gene transcription, protein expression, or actual pathway activity. Multi-omics strategies including transcriptomics (RNA-seq), proteomics, and targeted gene-knockout assays are required to establish causal links between gene repertoire and phenotypic function.

Furthermore, all tests were short-term static batch experiments performed at 200 rpm, generating convection-dominated mass-transfer conditions that favor high utilization of adsorption sites. Practical scenarios such as ecological ditches and constructed wetlands are governed by slow diffusive flow (<0.1 m s^−1^). Under such hydrodynamic conditions, inner adsorption sites may not become saturated before contaminant breakthrough, which means the maximum adsorption capacity obtained in the static-batch system may not be fully realized under field conditions. Hence, the quantified apparent contribution values are only applicable to short-term static conditions and cannot represent long-term performance under continuous water scouring, variable environmental factors, or dissolved-organic-matter fouling.

Although BET and FTIR measurements suggest minor impacts of autoclaving on pore-scale and broad functional-group features [40,41,42], quantitative analysis of oxygen-containing surface functional groups was not performed. Small systematic errors originating from subtle thermally induced surface modification therefore cannot be fully excluded. In addition, the composite material was prepared under a single set of experimental conditions in this study. EM dosage, immobilization duration, and solid–liquid ratio jointly shape pore evolution and microbial-loading efficiency, and the optimal preparation parameters for practical application still require further screening via single-factor and orthogonal experimental designs.

Overall, the core mechanistic interpretation drawn from this work is plausible under laboratory conditions. Nevertheless, the performance metrics reported herein require further systematic validation through co-ion interference tests, dissolved-organic-matter tolerance assays, dynamic column simulation, and field-scale pilot trials before large-scale practical application for agricultural-pollution remediation can be realized.

## 5. Conclusions

The EM-modified corn straw biochar fabricated in this study achieved an ammonium removal capacity of 1.322 mg/g, approximately 116% higher than that of pristine biochar (0.613 mg/g). Sterilization control experiments revealed that physicochemical adsorption served as the dominant removal pathway, while biodegradation played an auxiliary role. The enhanced adsorption performance stemmed from pore structure optimization: the mesoporous specific surface area increased by 129.4%, and the shift of pore-size distribution toward the mesopore range effectively reduced mass transfer resistance. Metagenomic analysis identified complete functional genes involved in the HN-AD pathway, including *amoA*, *hao*, *napA*, *nirK*, *norB*, and *nosZ*, suggesting the genetic potential of the EM consortium for ammonium degradation. Thermodynamic analysis indicated a decreased adsorption energy barrier after modification. This work provides mechanistic insights into the potential synergistic adsorption–biodegradation effect of biochar–microbe composites under simplified laboratory conditions. It should be noted that the apparent contribution of adsorption and biodegradation is conditional. Since all experiments were carried out in deionized-water-based synthetic solutions, further systematic validations including co-ion interference tests, dynamic column simulation, and field-scale trials are required before practical application in real farmland-drainage treatment.

## Figures and Tables

**Table 1:** Key enzyme genes involved in nitrogen metabolism and ARO abundance.

Gene Name	Database Accession Number	Functional Description	EC Number	Total Reads
*hao*	*K10535*	Hydroxylamine oxidoreductase catalyzes the conversion of hydroxylamine to nitrites.	1.7.2.6	1134
*norB*	*K04561*	Nitric oxide reductase catalyzes NO → N_2_O.	1.7.2.5	1470
*amoA*	*K10944*	catalytic oxidation of ammonia to hydroxylamine	1.14.99.39	980
*napA*	*K02567*	catalytic reduction of nitrate to nitrite	1.9.6.1	1223
*nirK*	*K00368*	catalytic reduction of nitrite to nitric oxide	1.7.2.1	1440
*nosZ*	*K00376*	catalytic reduction of nitrous oxide to nitrogen	1.7.2.4	1432
*patA*	ARO:3000021	ABC efflux pump ATP-binding protein	—	79,034
*patB*	ARO:3000025	ABC efflux pump permease component	—	22,704

**Table 2:** Adsorption kinetic models for biochar.

	Pseudo-First-Order Kinetic Model			Pseudo-Second-Order Kinetic Model			Intra-Particle Diffusion Model	
	*K*_1_	*q_e_* (mg/g)	*R* ^2^	*K*_2_	*q_e_* (mg/g)	*R* ^2^	*K_id_*	*R* ^2^
Primitive biochar	0.062	0.549	0.988	0.006	0.613	0.999	0.53	0.999
Modified biochar	0.133	1.248	0.966	0.01	1.322	0.987		
Sterilized modified biochar	0.134	1.148	0.963	0.013	1.212	0.989	0.95	0.997
Raw biochar was mixed with free EM bacteria	0.057	0.617	0.968	0.0065	0.692	0.995		

Note: *K_id_* values for unsterilized modified biochar and physical mixture groups are not applicable (N/A), as the intra-particle diffusion model requires pure adsorption systems without microbial interference.

**Table 3:** Pore structure of biochar, isothermal adsorption, thermodynamic experimental parameters.

	Pore Structure Parameters	Numerical Value	Langmuir Model Parameters	Numerical Value	Freundlich Model Parameters	Numerical Value	Thermodynamic Parameters	Numerical Value
Sterilized modified biochar	S_BET_ (m^2^/g)	14.906	*q_max_* (mg/g)	1.215	*K_F_* (mg/g)(L/mg)^1/^*^n^*	0.21	*ΔG^⊖^* (298.15 K) (kJ/mol)	8.574
	S_BJH,Ads_ (m^2^/g)	16.149	*K_L_* (L/mg)	15.7	1/n	1.037	*ΔH^⊖^* (kJ/mol)	9.393
	S_BJH,Des_ (m^2^/g)	12.786	*R* ^2^	0.946	*R* ^2^	0.959	*ΔS^⊖^* (J/mol⋅K)	1.986
	Average poresize (nm)	5.6144						
Primitive biochar	S_BET_ (m^2^/g)	26.582	*q_max_* (mg/g)	0.73	*K_F_* (mg/g)(L/mg)^1/^*^n^*	0.147	*ΔG^⊖^* (298.15 K) (kJ/mol)	10.693
	S_BJH,Ads_ (m^2^/g)	7.041	*K_L_* (L/mg)	0.139	1/n	1.023	*ΔH^⊖^* (kJ/mol)	12.240
	S_BJH,Des_ (m^2^/g)	11.764	*R* ^2^	0.956	*R* ^2^	0.985	*ΔS^⊖^* (J/mol⋅K)	5.195
	Average pore size (nm)	5.1243						

## Data Availability

The raw experimental data supporting the findings of this study are available on reasonable request from the corresponding author.
